# Risks and seasonal pattern for mortality among hospitalized infants in a conflict-affected area of Pakistan, 2013-2016. A retrospective chart review.

**DOI:** 10.12688/f1000research.19547.1

**Published:** 2019-06-24

**Authors:** Babette van Deursen, Annick Lenglet, Cono Ariti, Barkat Hussain, Jaap Karsten, Harriet Roggeveen, Debbie Price, Jena Fernhout, Ahmed Abdi, Antonio Isidro Carrion Martin

**Affiliations:** 1 Médecins Sans Frontières - Operational Centre Amsterdam, Amsterdam, The Netherlands; 2 Radboud University Nijmegen Medical Centre, Nijmegen, The Netherlands; 3 Cardiff University Medical School, Cardiff, UK; 4 Pediatric Division Head Quarter-Ministry of Health, Balochistan, Pakistan; 5 Médecins Sans Frontières - Pakistan, Balochistan, Pakistan; 6 Médecins Sans Frontières - UK, London, UK

**Keywords:** Infant mortality; Epidemiology; Seasonal pattern; Pakistan; MSF

## Abstract

**Background:** In recent years, Médecins Sans Frontières has observed high mortality rates among hospitalized infants in Pakistan. We describe the clinical characteristics of the infants admitted between 2013 and 2016 in order to acquire a better understanding on the risk factors for mortality.

**Methods:** We analyzed routinely collected medical data from infants (<7 months) admitted in Chaman and Dera Murad Jamali (DMJ) hospitals. The association between clinical characteristics and mortality was estimated using Poisson regression.

**Results:** Between 2013 and 2016, 5,214 children were admitted (male/female ratio: 1.60) and 1,178 (23%) died. Days since admission was associated with a higher risk of mortality and decreased with each extra day of admission after seven days. The first 48 hours of admission was strongly associated with a higher risk of mortality. A primary diagnosis of tetanus, necrotizing enterocolitis, prematurity, sepsis and hypoxic-ischemic encephalopathy were strongly associated with higher rates of mortality. We observed an annual peak in the mortality rate in September.

**Conclusions:** The first days of admission are critical for infant survival. Furthermore, the found male/female ratio was exceedingly higher than the national ratio of Pakistan. The observed seasonality in mortality rate by week has not been previously reported. It is fully recommended to do further in-depth research on male/female ratio differences and the reasons behind the annual peaks in mortality rate by week.

## List of abbreviations:

95%CI    95% Confidence Intervals

aRR       Adjusted Rate Ratio

DHS      Demographic and Health Survey

DMJ      Dera Murad Jamali

HIE       Hypoxic-ischemic Encephalopathy

MoH      Ministry of Health

MSF      Médecins Sans Frontières

MSF-OCA   Médecins Sans Frontières Operational Centre Amsterdam

NEC      Necrotizing Enterocolitis

RR      Rate Ratio

## Introduction

As part of the Millennium Development Goals, the under-five mortality (U5M) should be reduced by two-thirds from 1990 to 2015 ^1, 2^. In 2015, the U5M was reportedly 47 per 1,000 live births in low resource settings compared to 6 per 1,000 live births in high resource countries ^2^. Mortality in children under five is still high in Southern Asia compared to other regions. In Pakistan, the Demographic and Health Survey (DHS) of 2012-13 showed that mortality among neonates and infants was still one of highest in South Asia. Especially in the Balochistan region, where the neonatal mortality was 55 deaths per 1,000 live births and under-five mortality was 111 deaths per 1,000 live births ^3^.

**Figure 1.**
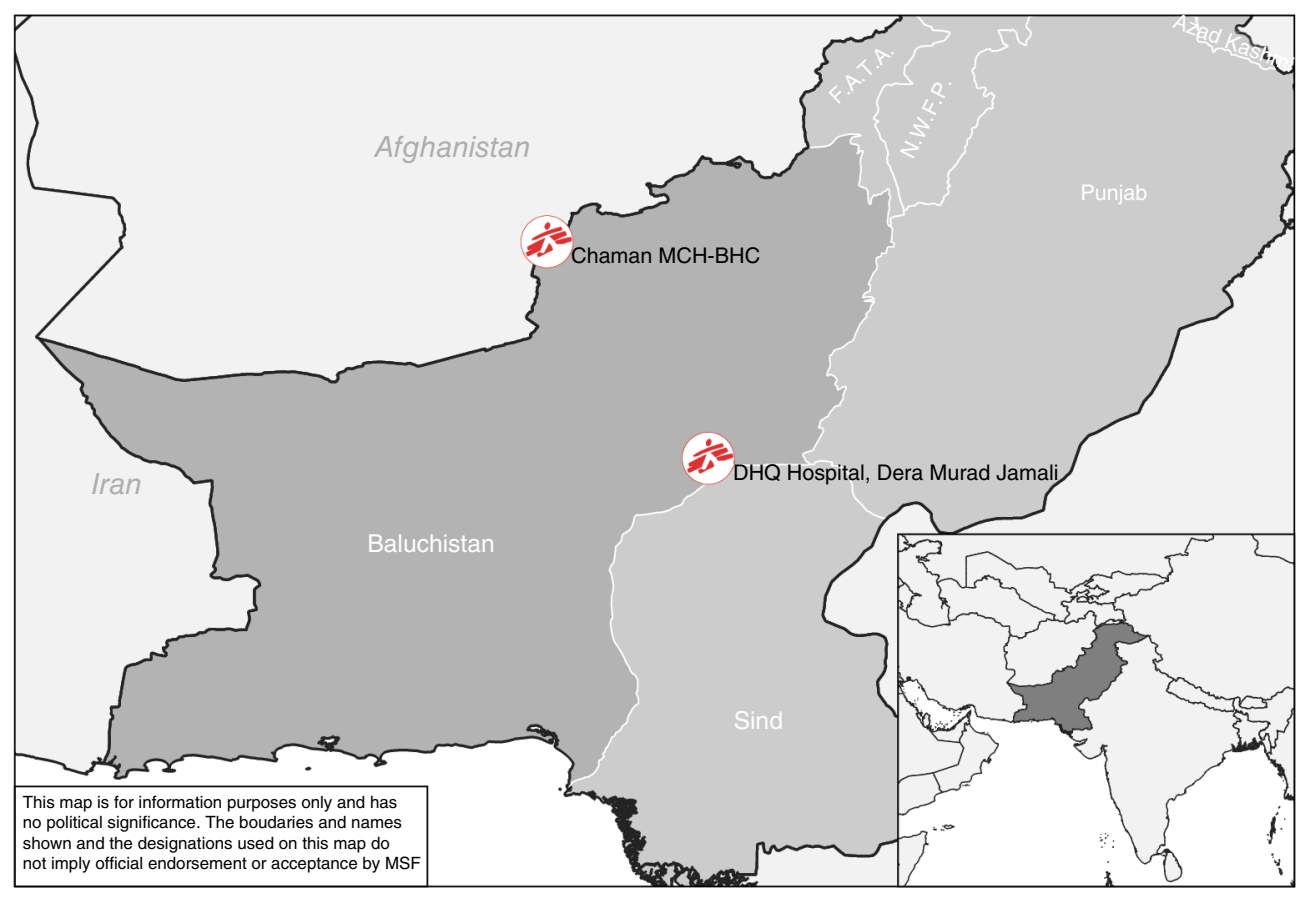
Projects of Médecins Sans Frontières (MSF) in Pakistan. Author: MSF UK Data Source: Natural Earth; MSF

Médecins Sans Frontières works in cooperation with the Ministry of Health (MoH) in two hospitals in the Balochistan province of Pakistan ^4^. Balochistan is an unstable and vulnerable province due to historical disputes between different (ethnic) groups ( Figure 1) ^5^. One of the hospitals is located in Chaman, in the north of Balochistan near the Afghan border. The Chaman project offers services to the residents, to Afghan refugees and also to Afghans crossing the border in search of medical services. The second hospital is located in the southern region of Balochistan in Dera Murad Jamali (DMJ). This hospital offers services to the residents of Nasirabad and Jafarabad districts.

In recent years, MSF has observed high in-hospital mortality among neonates and infants in both Chaman and DMJ hospitals. Since 2010, the recorded monthly neonatal mortality (neonatal deaths amongst all neonatal exits from the neonatal department) has exceeded 25% in both hospitals (MSF unpublished data). In order to better understand the causes for mortality and provide recommendations for clinical care, we aimed to describe the clinical characteristics of the infants under seven months admitted between 2013 and 2016 Chaman and DMJ hospitals.

## Methods

### Design

This study consisted of secondary retrospective analysis of routinely collected data in the neonatal departments of DMJ and Chaman hospitals. The clinical characteristics available for analysis were: sex, date of admission and exit (discharge or death), primary diagnosis, and outcome (i.e. death or alive). We were only able to classify patients in two age groups, under seven months and greater or equal to seven months. Thus, no specific ages were available for more refined analyses on age of infants.

### Study population and setting

All children younger than seven months old admitted to the DMJ and Chaman hospital between 2013 and 2016 were included in this study. Patients, for whom crucial data was missing such as exit date and primary diagnosis, were excluded from this study.

### Statistical analysis

All data was extracted and anonymized before the analysis. We calculated an exposure time (person days) for each patient by subtracting their date of admission from the date of discharge. The primary diagnosis was categorized into sepsis, neonatal tetanus, necrotizing enterocolitis (NEC), prematurity, hypoxic-ischemic encephalopathy (HIE), maternal-fetal infection, respiratory syndromes and other. Those were chosen due to the high frequency of reported diagnosis and other contained the remaining diagnoses.

The mortality rate by month was calculated with their corresponding 95% CI and a two-months-moving-average was used to explore seasonality.

We used Poisson regression to calculate the unadjusted Rate Ratios (RRs) with their respective 95% confidence intervals (95% CI) and p-values to assess the association of each possible risk factor with the outcome of in-hospital mortality. For the primary diagnosis, we used respiratory syndromes as the reference group as it had lowest mortality rate and a sufficient number of events. A Poisson multivariable regression model was used to calculate adjusted RRs (aRR) with their corresponding 95% CI and p-values. Each patient’s exposure time was split into single day periods and this elapsed time variable was included in the Poisson models to estimate the mortality rate, rate ratios, 95% CIs and p-values for each day since admission.

The data cleaning and manipulation was done using Excel 2010, and data analysis was done using Stata IC 14 ^6^. We did not merge the datasets from the two hospitals, because it would be more useful for the teams as the two hospitals contexts were different and presenting merged results may have diluted the specificities in the particular situations each team faces.

***Ethical consideration:*** This was a retrospective post-hoc analysis of routinely collected clinical data; therefore, it was exempted from MSF ethical board review. The MSF-OCA medical director gave his approval for this analysis. The data in the utilized datasets did not contain individual identifiers and it was password protected and only accessible by the research team.

## Results

### Chaman

Between 2013 and 2016, there were 2,551 infants admitted in the Inpatient Department (IPD) of Chaman, 563 (22%) of them died ( Table 1). There were more males admitted (n=1,573) than females (n=977); the male to female ratio was 1.61. The diagnosis NEC and HIE were responsible for the highest mortality rates ( Table 2).

**Table 1.** Patient characteristics and analysis results of the infants (<7 months) who were admitted between 2013 and 2016 in the Inpatient Department (IPD) of Chaman.

Characteristics	Admitted (n=2,551)	Died (n=563)	Unadjusted Rate Ratios [95%CI]	P-value	Adjusted Risk Ratios [95%CI]	P-value
**Sex** *Female* Male *Unknown*	977 1,573 1	211 (21.6%) 351 (22.3%) 1 (100%)	0.95 [0.80-1.13] *Ref.* -	0.564 - -	0.94 [0.79-1.12] *Ref.* -	0.505
**Year of admission** *2013* *2014* *2015* *2016*	511 682 635 723	111 (21.7%) 176 (25.8%) 138 (21.7%) 138 (19.1%)	*Ref.* 1.44 [1.13-1.82] 1.21 [0.94-1.56] 1.12 [0.87-1.44]	0.019	*Ref.* 1.56 [1.14-2.14] 1.30 [0.98-1.73] 1.28 [0.95-1.71]	0.048
**Month of admission** *January* *February* *March* *April* *May* *June* *July* *August* *September* *October* *November* *December*	240 219 220 206 189 210 202 221 208 215 228 193	54 (22.5%) 47 (21.5%) 50 (22.7%) 42 (20.4%) 30 (15.9%) 38 (18.1%) 48 (23.8%) 46 (20.8%) 73 (35.1%) 47 (21.9%) 42 (18.4%) 46 (23.8%)	*Ref.* 1.05 [0.71-1.56] 1.02 [0.70-1.50] 0.97 [0.65-1.45] 0.71 [0.45-1.11] 0.83 [0.55-1.26] 1.18 [0.80-1.74] 1.02 [0.69-1.51] 2.05 [1.44-2.92] 1.03 [0.70-1.52] 0.86 [0.57-1.28] 1.36 [0.92-2.01]	<0.001	*Ref.* 0.83 [0.56-1.24] 0.95 [0.64-1.39] 0.81 [0.53-1.24] 0.59 [0.38-0.92] 0.62 [0.41-0.96] 0.87 [0.58-1.30] 0.75 [0.50-1.12] 1.38 [0.95-2.02] 0.87 [0.58-1.28] 0.76 [0.51-1.14] 1.23 [0.83-1.82]	0.001
**Number of days since admission** *0 days* *1 day* *2 days* *3 days* *4 days* *5 days* *6 days* *7 days* *8 days* *>8 days*	226 245 336 347 339 212 181 134 90 441	85 (37.6%) 127 (51.8%) 84 (25.0%) 49 (14.1%) 63 (18.6%) 53 (25.0%) 38 (21.0%) 25 (18.7%) 7 (7.8%) 32 (7.3%)	2.75 [1.83-4.13] 4.60 [3.12-6.78] 3.63 [2.42-5.45] 2.64 [1.69-4.13] 4.49 [2.94-6.88] 4.73 [3.05-7.34] 4.32 [2.70-6.92] 3.57 [2.11-6.02] 1.20 [0.53-2.73] *Ref.*	<0.001	3.11 [2.06-4.68] 5.20 [3.52-7.69] 4.03 [2.67-6.08] 2.93 [1.87-4.59] 4.91 [3.20-7.53] 5.20 [3.34-8.08] 4.72 [2.94-7.56] 3.85 [2.28-6.50] 1.28 [0.57-2.91] *Ref.*	<0.001
**Bed occupancy rate** *0 – 85 %* *> 85 %*	949 1,602	225 (23.7%) 338 (21.1%)	*Ref.* 0.95 [0.80-1.12]	- 0.512	*Ref.* 0.85 [0.67-1.08]	0.187
**Primary diagnosis** *Sepsis* *NEC* *HIE* *Prematurity* *Respiratory syndromes* *Other*	803 18 437 273 468 552	190 (23.7%) 15 (83.3%) 142 (32.5%) 118 (43.2%) 43 (9.2%) 55 (10.0%)	1.99 [1.43-2.77] 5.47 [3.04-9.84] 3.01 [2.14-4.24] 2.71 [1.91-3.85] *Ref.* 1.11 [0.74-1.65]	<0.001	2.11 [1.50-2.98] 5.79 [3.20-10.48] 3.25 [2.29-4.61] 3.41 [2.39-4.86] *Ref.* 1.16 [0.78-1.73]	<0.001 -

**Table 2.** Mortality rates per 1,000 person days (95%CI) for the primary diagnoses in Chaman.

Primary diagnosis	Mortality rates per 1,000 person days (95% CI)
**NEC** **Sepsis** **HIE** **Prematurity** **Respiratory syndromes** **Other**	114.50 (69.03 – 189.93) 41.59 (36.08 – 47.95) 63.05 (53.49 – 74.33) 56.79 (47.41 – 68.01) 20.93 (15.53 – 28.23) 23.17 (17.79 – 30.18)

NEC - necrotizing enterocolitis, HIE - hypoxic-ischemic encephalopathy

In the adjusted analysis ( Table 1), the number of days since admission was strongly associated with a higher risk of mortality when compared to day eight or more days since admission. The highest risks for mortality were observed during the first five days since admission and decreased after that with each extra day of admission: at day 5 aRR=5.20 (95% CI: 3.34-8.08) and at day 8 aRR=1.28 (95% CI: 0.57-2.91). Furthermore, some primary clinical diagnoses were also associated with mortality compared to a diagnosis of respiratory syndromes, namely: NEC (aRR= 5.79; 95% CI: 3.20-10.48), prematurity (aRR=3.41; 95% CI: 2.39-4.86), HIE (aRR=3.25; 95% CI: 2.29-4.61) and suspected clinical sepsis (aRR=2.11; 95% CI: 1.50-2.98).

### DMJ

Between 2013 and 2015, there were 2,663 infants admitted in the IPD and 23% (n=615) died during their admission ( Table 3). More males (n=1,636) were admitted than females (n=1,207); the male/female ratio was 1.59. The primary diagnosis tetanus, NEC, HIE and sepsis had the highest mortality rates ( Table 4).

**Table 3.** Patient characteristics and analysis results of the infants (<7 months) who were admitted between 2013 and 2016 in the Inpatient Department (IPD) of Dera Murad Jamali (DMJ).

Characteristics	Admitted (n=2,663)	Died (n=615)	Unadjusted Rate Ratio [95%CI]	P-value	Adjusted Rate Ratio [95%CI]	P-value
**Sex** *Female* *Male*	1,027 1,636	241 (23.5%) 374 (22.9%)	1.05 [0.89-1.24] *Ref.*	0.542	1.10 [0.93-1.30] *Ref.*	0.254
**Year of admission** *2013* *2014* *2015* *2016*	743 742 595 583	147 (19.8%) 170 (22.9%) 158 (26.6%) 140 (24.0%)	*Ref.* 1.04 [0.83 -1.30] 0.95 [0.76-1.19] 0.87 [0.69-1.10]	0.446	*Ref.* 0.91 [0.71-1.16] 0.94 [0.74-1.19] 0.80 [0.63-1.03]	0.384
**Month of admission** *January* *February* *March* *April* *May* *June* *July* *August* *September* *October* *November* *December*	241 228 213 198 181 166 217 238 273 268 251 189	53 (22.0%) 52 (22.8%) 43 (20.2%) 40 (20.2%) 47 (26.0%) 29 (17.5%) 47 (21.7%) 58 (24.4%) 80 (29.3%) 72 (26.9%) 57 (22.7%) 37 (19.6%)	*Ref.* 1.01 [0.69-1.48] 0.90 [0.61-1.35] 0.84 [0.56-1.27] 0.96 [0.65-1.43] 0.63 [0.40-0.99] 0.93 [0.63-1.37] 0.96 [0.66-1.40] 1.28 [0.90-1.81] 1.13 [0.79-1.61] 0.87 [0.60-1.26] 0.82 [0.54-1.25]	0.135	*Ref.* 0.97 [0.66-1.43] 0.85 [0.56-1.28] 0.80 [0.52-1.21] 1.01 [0.66-1.53] 0.56 [0.37-0.93] 0.93 [0.62-1.39] 0.81 [0.55-1.18] 1.15 [0.80-1.64] 1.06 [0.74-1.53] 0.95 [0.64-1.39] 0.90 [0.59-1.38]	0.300
**Number of days since admission** *0 days* *1 day* *2 days* *3 days* *4 days* *5 days* *6 days* *7 days* *8 days* *>8 days*	179 366 314 307 262 184 182 157 106 606	114 (63.7%) 192 (52.5%) 110 (31.9%) 54 (17.6%) 51 (19.5%) 27 (14.7%) 22 (12.1%) 14 (8.9%) 8 (7.6%) 33 (5.5%)	5.15 [3.48-7.62] 10.17 [7.00-14.79] 6.29 [4.23-9.37] 4.06 [2.62-6.29] 4.66 [2.99-7.25] 2.90 [1.74-4.84] 2.87 [1.67-4.94] 2.24 [1.19-4.19] 1.51 [0.69-3.27] *Ref.*	<0.001	6.68 [4.48-9.95] 13.38 [9.14-19.59] 8.26 [5.51-12.37] 5.25 [3.37-8.17] 5.85 [3.74-9.14] 3.55 [2.12-5.95] 3.40 [1.97-5.86] 2.61 [1.39-4.90] 1.72 [0.79-3.73] *Ref.*	<0.001
**Bed occupancy rate** *0 – 85 %* *> 85 %*	1,309 1,354	294 (22.5%) 321 (23.7%)	*Ref.* 1.09 [0.93-1.28]	0.288	*Ref.* 0.95 [0.76-1.19]	0.655
**Primary diagnosis** *Sepsis* *Neonatal tetanus* *NEC* *Prematurity* *HIE* *Maternal-fetal infection* *Respiratory syndromes* *Other*	536 110 7 293 476 50 604 587	152 (28.3%) 76 (69.1%) 5 (71.4%) 101 (34.5%) 147 (30.9%) 14 (28.0%) 48 (8.0%) 72 (12.3%)	2.77 [2.00-3.83] *.* 4.98 [3.47-7.14] 9.69 [3.86-24.33] 1.92 [1.36-2.70] 3.52 [2.54-4.88] 2.02 [1.11-3.66] *Ref.* 1.65 [1.15-2.38]	<0.001	3.43 [2.47-4.76] 8.50 [5.88-12.29] 11.65 [4.56-29.76] 3.32 [2.33-4.72] 4.13 [2.97-5.75] 3.26 [1.77-6.01] *Ref.* 1.74 [1.21-2.52]	<0.001

NEC - necrotizing enterocolitis, HIE - hypoxic-ischemic encephalopathy

**Table 4.** Mortality rates per 1,000 person days (95%CI) for the primary diagnoses in Dera Murad Jamali (DMJ).

Primary diagnosis	Mortality rates per 1,000 person days (95% CI)
**Sepsis** **Neonatal tetanus** **NEC** **Prematurity** **HIE** **Maternal-fetal infection** **Respiratory syndromes** **Other**	46.06 (39.29 – 54.00) 82.88 (66.19 – 103.77) 161.290 (67.13 – 387.51) 31.92 (26.27 – 38.80) 58.63 (49.88 – 68.92) 33.57 (19.88 – 56.69) 16.65 (12.55 – 22.09) 27.55 (21.87 – 34.71)

NEC - necrotizing enterocolitis, HIE - hypoxic-ischemic encephalopathy

In the adjusted analysis ( Table 3), the number of days since admission was strongly associated with death and with each extra day in the hospital this risk decreased when compared to day eight or more since admission: at day one aRR=13.38 (95% CI: 9.14-19.59) and at day eight aRR=1.72 (95% CI:0.79-3.73). In addition, patients with one of the following diagnosis had an increased risk for death versus a diagnosis of respiratory syndrome: suspected neonatal tetanus (aRR=8.50; 95% CI: 5.88-12.29), NEC (aRR=11.65; 95% CI: 4.56-29.76), HIE (aRR=4.13; 95% CI: 2.97-5.75), sepsis (aRR=3.43; 95% CI: 2.47-4.76) and prematurity (aRR=3.32; 95% CI: 2.33-4.72).

### Seasonality

**Figure 2.**
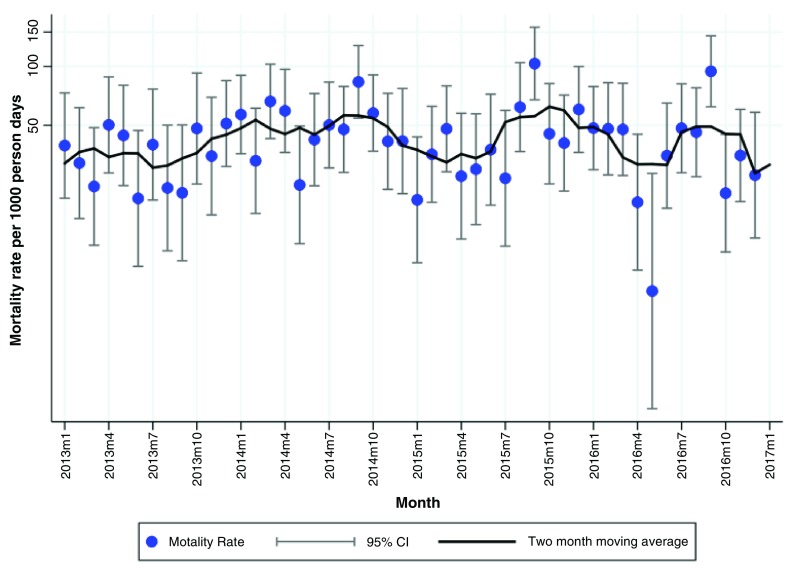
Monthly mortality rate (per 1,000-person days) and a 95% confidence interval and two months moving average smoothed morality rates for Chaman.

**Figure 3.**
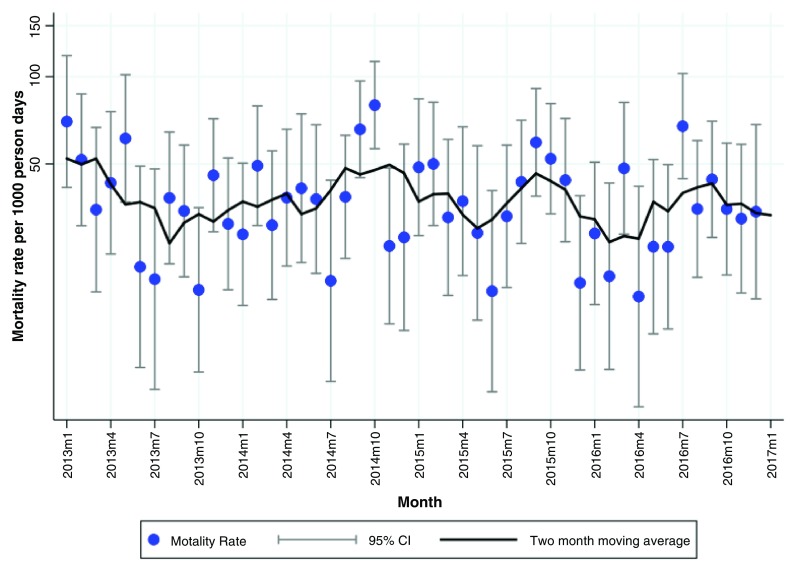
Monthly mortality rate (per 1,000-person days) and a 95% confidence interval and two months moving average smoothed morality rates for Dera Murad Jamali (DMJ).

We observed an annual peak in the mortality rate between July and October in both hospitals with the mortality rate peaking in September. On average, the two-months-moving-average of the mortality rate was 40 per 1,000 person days (exposure time) for Chaman. The annual peaks in Chaman had a mortality rate that varied between 40-100 per 1,000 person days ( Figure 2). For DMJ, the two-months-moving average was on average around 30 per 1000 person days. The mortality rates in the annual peaks varied between 40-70 per 1,000 person days ( Figure 3). There was considerable month to month variation in mortality rates as can be observed from the 95% CIs shown in Figure 2 and Figure 3. Smoothing these crude rates using a two-month moving average still showed some evidence of an annual peaks.

## Discussion

We found that the mortality in children under seven months age was 23% in two hospitals in Balochistan province in Pakistan between 2013 and 2016. In both hospitals, the number of days since admission was strongly associated with death: infants were more likely to die during the first 48 hours of admission, with the greatest risk at day 1 (24 to 48 hours since admission). This differs with other studies which suggest that the longer the stay in the hospital, the higher the chance to develop nosocomial infection leading to death ^7^. Also, a longer stay in the hospital has been associated to severity of illness ^8^.

We assume that the high mortality rate is not necessarily due to the care that MSF provides, but due to late presentation and critical state of the majority of patients in both hospitals. Anecdotally, MSF staff report that the patients are often treated with multiple antibiotics from other private health care centers. Neonatal care in private hospitals is expensive and when the financial resources of families are exhausted, parents seek help in MSF hospitals. It is known that in developing countries the private sector is preferred over public sector ^9^, however, the provided service is debatable. We did not have any information on date of on onset of symptoms to be able to evaluate whether late presentation played a role in high mortality rates early on in their admission. Nevertheless, literature states that infant survival is lower when there is a delay in seeking health care ^10^. Improving the health care seeking behavior will increase infant survival.

The male/female ratio in our study (1.60) was higher to the one found in the 2012-2013 DHS in Pakistan (1.04) ^3^. This difference could be explained by son preference which has been described before in South Asia ^11^ and/or by a reduced healthcare seeking behavior for girls by the parents in this region ^1, 12^, but we cannot be sure as this goes beyond the objective of the study.

We identified a seasonal pattern in the monthly mortality rate (per 1,000 person days) in hospitalized infants in Chaman and DMJ around September ( Figure 2 and Figure 3). To our knowledge, the observed seasonal pattern has not been mentioned in the literature before. Two studies conducted in South Asia (Nepal and Bangladesh) among local populations have previously mentioned a seasonal pattern in mortality, but they found different seasonality patterns. In Nepal, the neonatal mortality rate was the highest between April and October, but the highest peak was observed in August (13). In Bangladesh, the peak of neonatal deaths was observed in November and for 1–4 year olds in the hot-wet season (July-September) ^13, 14^. However, the seasonality in mortality could be explained by seasonality of diseases. A study in Northern Pakistan showed that the highest prevalence of malaria parasites was found in infants after monsoon season (September-November) ^15^. This similar peak (September-November) was also observed for dengue cases among admitted patients in the districts Shangla and Buner in Northern Pakistan ^16^. In our IPD data there were no dengue diagnosis registered and the number of malaria diagnosis was very low (total 2013-2016: two in Chaman and 203 in DMJ), and did not show a seasonal pattern with increase in numbers in September. 

The main limitation of this study was that it was a retrospective chart review, so there was no control in the use of data collection tools and on disease coding. We could also not explore the impact of infant age on mortality during this study. Despite the limitations, our study had the great strength of the large amount of observations around inpatient pediatric patients in this part of Pakistan, therefore the data included and the findings are highly relevant.

## Conclusion

The first two days of admission are critical for infant survival in the MSF hospitals in Balochistan, an underserved area of Pakistan in terms of health care. We found an annual seasonal pattern in mortality rate by week and a male/female ratio in that was higher than the known male/female ratio of Pakistan. Further investigations are needed to establish i) if the cause of this male/female ratio differences is gender differences in access to care or an actual difference in burden of disease by gender and ii) the reasons behind these annual peaks in mortality rate by week. We recommend targeting efforts on increasing quality of care during the first days of admission and to allocate resources accordingly, and also taking into account the seasonal pattern.

## Ethical considerations

This was *a posteriori* analysis of routinely collected clinical data; therefore, it was exempted from ethical board review. The MSF-OCA medical director gave his approval for this analysis. The data in the utilized datasets did not contain individual identifiers and it was password protected and only accessible by the research team.

## Data availability

### Underlying data

The nature of MSF operations and target populations are such that data collected often involves highly Sensitive Data. Recipients, who wish to access any MSF Datasets that include Personal Data and/or Human Samples, must secure ethical clearance from competent ethical authorities and of MSF ERB. If a reader wants to access the data he/she can find more information in the MSF Data Sharing Policy ( http://hdl.handle.net/10144/306501).
