# Supplementary material for: Risks and seasonal pattern for mortality among hospitalized infants in a conflict-affected area of Pakistan, 2013-2016. A retrospective chart review
Source: F1000Res. 2019 Jun 24;8:954. [Version 1] doi: 10.12688/f1000research.19547.1 (PMC6733381; doi:10.12688/f1000research.19547.1)
Supplement: Peer review history

# Peer review: Reviewer response for version 1

**DOI:** 10.5256/f1000research.21431.r51353

**Published:** 2019-09-06

**Authors:** Sharif Ismail

**Affiliations:** 1 Department of Primary Care and Public Health, Imperial College London, London, UK; 2 Department of Global Health and Development, London School of Hygiene and Tropical Medicine, London, UK

Thank you for the opportunity to review this paper, which presents data from two MSF hospitals in Pakistan to explore clinical factors associated with mortality among children admitted to these facilities. The analysis of seasonality in particular is an interesting and novel feature of this analysis although it is notable that differences between the two sites in this regard are not commented on in the manuscript.

 There are some important ways in which the work could have been strengthened. For example:

1. The choice of a 7-month age cut off in defining the patient group is unusual as it corresponds neither to traditional definitions of the neonatal nor infant periods. It would have been helpful to know exactly why it would possible only according to the 7-month cut off and to have read a clearer explanation of the implications of this limitation for the analysis - and more on data limitations overall would have been useful.
2. Tables 2 and 4 aggregate diagnostic categories differently - why is this? Were no cases reported against the other categories? Separating out categories in the same way would have aided qualitative comparisons between the two sites.
3. The authors state that a pooled analysis across the two facilities was not practically useful to the teams. This is fine - but it would then have been helpful to include more data (if available) to test to account for observed differences - especially in mortality within the first 48 hours of admission. Were any triage assessment measures on admission available to include?

I confirm that I have read this submission and believe that I have an appropriate level of expertise to confirm that it is of an acceptable scientific standard.

# Peer review: Reviewer response for version 1

**DOI:** 10.5256/f1000research.21431.r51351

**Published:** 2019-07-26

**Authors:** Eva Leidman

**Affiliations:** 1 Center for Global Health, Centers for Disease Control and Prevention, Atlanta, GA, USA

The authors use routine data from two MSF hospitals in Pakistan to explore factors associated with in-hospital mortality among infants and neonates. As such the manuscript presents interesting analysis of an important research question. The authors nicely characterize the benefits and limitations of the retrospective data (e.g., lack of age data) used in the presented manuscript.

 The manuscript would benefit from further details pertaining to the statistical analysis, as well as justification of the model selection. Additional details should include analysis performed to assure the assumptions of the Poisson model hold (e.g., measure of dispersion), and clarity on how the offset was modeled.

 Additionally, as the key finding of the manuscript relates to differences in mortality associated with days since admission, (descriptive) analysis comparing characteristics of children with exits in the first 48 hours to the reference would be strengthen the manuscript, and help explore implications of the regression findings. Finally, global tests for month of admission (seasonality) appear only significant for Chaman (not DMJ). Discussing this distinction would also strengthen the manuscript.

I confirm that I have read this submission and believe that I have an appropriate level of expertise to confirm that it is of an acceptable scientific standard.

# Peer review: Reviewer response for version 1

**DOI:** 10.5256/f1000research.21431.r50282

**Published:** 2019-07-08

**Authors:** Babar Tasneem Shaikh

**Affiliations:** 1 Health Services Academy, Islamabad, Pakistan

This is an interesting approach to study such data in retrospective fashion. Findings on most common newborn or infants’ illnesses and their outcomes (discharge or mortality) in Pakistan are well known and covered under various national surveys also. PDHS and MICS are two such examples besides various individual and organizational work published in recent years.

 The paper under consideration has used PDHS 2012-13 as a baseline and has attempted to make a case for two sites in Balochistan. The main thrust is that province under-performs vis-à-vis national statistics. This is not a new revelation and is a well-known and documented fact for many decades. With new PDHS 2017-18 released, this paper becomes redundant. Secondly, the causes of hospital admission or diagnoses found in infants are also chronologically known. Only thing which could have made this paper more interesting is adding the work of MSF which is being done at these two sites to address the issue of neonatal morbidity and mortality and the details of interventions which are being introduced at hospital or community level and then results shown with a significant difference made.

I confirm that I have read this submission and believe that I have an appropriate level of expertise to confirm that it is of an acceptable scientific standard, however I have significant reservations, as outlined above.
